# NUT carcinoma of the submandibular gland: A case report

**DOI:** 10.1002/cnr2.1900

**Published:** 2023-09-08

**Authors:** Simone Rota, Pasquale Quattrone, Giovanni Centonze, Gianpaolo Dagrada, Arianna Ottini, Elena Colombo, Imperia Nuzzolese, Giuseppina Calareso, Marzia Franceschini, Nicola Alessandro Iacovelli, Federica Perrone, Elena Tamborini, Stefano Cavalieri

**Affiliations:** ^1^ Head and Neck Medical Oncology Department Fondazione IRCCS Istituto Nazionale dei Tumori di Milano Milan Italy; ^2^ Pathology Department Fondazione IRCCS Istituto Nazionale dei Tumori di Milano Milan Italy; ^3^ Radiology Department Fondazione IRCCS Istituto Nazionale dei Tumori di Milano Milan Italy; ^4^ Radiotherapy Department Fondazione IRCCS Istituto Nazionale dei Tumori di Milano Milan Italy; ^5^ Department of Oncology and Hemato‐oncology University of Milan Milan Italy

**Keywords:** chromosome 15q14, head and neck, NUT carcinoma, salivary glands, submandibular gland

## Abstract

**Background:**

NUT carcinoma (NUTc) is a rare and aggressive malignant epithelial tumor characterized by rearrangement of the NUT gene on chromosome 15q14.

**Methods:**

In this article, we present the fifth case worldwide of a young woman affected by a NUTc arising from a submandibular gland, presenting as a rapidly evolving mass. She underwent a right scialoadenectomy and received the initial diagnosis of high‐grade mucoepidermoid carcinoma. Due to evidence of local recurrence at magnetic resonance imaging 1 month later, a subsequent right radical neck dissection was performed. The patient then sought a second opinion at our cancer center and finally received the correct diagnosis of NUT carcinoma. Given the well‐known aggressive behavior of this neoplasm, as well as clinical and radiological features, she underwent adjuvant chemo‐radiation (intensity‐modulated radiotherapy + concurrent chemotherapy with cisplatin).

**Results:**

After a disease‐free interval of 2.6 months, a widespread metastatic disease led to rapid deterioration of performance status and patient death in a few weeks after metastatic onset.

**Conclusions:**

We presented a case of NUTc arising from salivary gland aiming to improve the knowledge of this rare malignancy. First, we pointed out that in the setting of rare tumors like salivary gland cancers, the diagnosis should be obtained by expert pathologists, and patients should be referred to tertiary cancer centers for their clinical management. Second, molecular profiling may help to identify possible druggable targets that may be exploited to treat patients suffering from this aggressive malignancy. Sharing the molecular data provided in this case will be useful for further research.

## 1 INTRODUCTION

NUT carcinomas are rare, poorly differentiated, aggressive malignant epithelial neoplasms characterized by rearrangement of the *NUT* (nuclear protein in testis) gene, located on chromosome 15q14. Approximately 70% of these cancers harbor the BRD4‐NUT fusion oncogene, resulting from a t(15;19) translocation. In the remaining 30% of cases, NUT rearrangements involve other genes, such as BRD3 and NSD3.^1^, ^2^, ^3^

NUT fusion proteins are responsible for the rapid and unregulated growth of the tumor mass.^3^ Indeed, in BRD4‐NUT fusion, the acetyl‐histone‐binding bromodomains of BRD4 combine NUT with chromatin. This leads to massive acetylation of large chromatin regions, activating a chain of reactions that leads to forced expression of critical oncoproteins (MYC, p63, MED24).^2^, ^3^

The highly aggressive characteristics of this malignancy have long been known. At diagnosis, lymph node or metastatic localizations are common. Despite treatment, only 20% of patients are alive 1 year after diagnosis, with a median survival of 6.7 months.^3^

Literature initially described these carcinomas as exclusive to childhood, but it is now proven that NUTc can affect people of all ages and sex. This could be explained considering the increased focus on the disease and the availability of cheaper diagnostic tests.^3^, ^4^

Moreover, NUTc can arise from different tissues and sites, so it cannot be classified based on the anatomic site of origin. At the same time, it was initially thought to start from the midline supradiaphragmatic airway locations and the mediastinum.^5^, ^6^ Nonetheless, many cases arising below the diaphragm and outside the midline have been described, including the bladder, pancreas, kidney, adrenal gland, and soft tissues.^7^, ^8^, ^9^, ^10^ Although the site of origin cannot be a criterion for classification, due to the frequent involvement of midline structures and the absence as well of in situ components when NUTc arise from an epithelium‐lined organ, it is hypothesized that these tumors arise from primitive neural crest cells.^5^

Head and neck NUTc prevalence is 39%, and the most frequent sites are the nasopharynx and oropharynx. Less frequently NUTc can arise from the major salivary glands, and an origin in the submandibular glands is extremely rare.^11^

Due to an overlap with other poorly differentiated carcinomas, morphological pathology is not diriment for diagnosis: this often leads to an initial misdiagnosis, commonly with poorly differentiated squamous cell carcinomas.^3^, ^5^

In this article, we describe the case of a young woman affected by a NUTc arising from the right submandibular gland, focusing on the importance of diagnosing and treating rare tumors in referral centers.

On the other hand, aiming to explore possible therapeutic targets, we will provide a molecular analysis of the carcinoma (retrieved with Oncomine Comprehensive Assay Plus), contextualizing our findings with the available literature data.

To our knowledge, this is the second reported case of NUTc arising from salivary glands where molecular profiling, PDL1, MSI, and tumor mutation burden (TMB) were explored. While this adds data to the preexisting literature, it also allows us to suggest the assessments be required to seek personalized therapies for these patients.

## 2 CASE PRESENTATION

**FIGURE 1:**
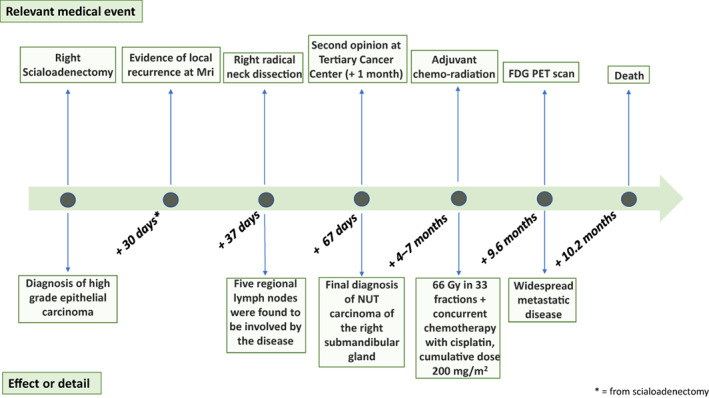
Timeline of patient's medical history. At the top of the figure, relevant medical events are represented, while at the bottom the consequent effects.

A 39‐year‐old woman underwent right scialoadenectomy after the persistence of a right submandibular lump for at least 8 months, unsuccessfully treated with anti‐inflammatory and steroid treatment (Figure 1). She was a former smoker (<10 pack‐years), and denied alcohol or drug abuse. Her medical history was unremarkable, except for a previous Hashimoto thyroiditis, which was well compensated with levothyroxine substitution. The physical examination was normal except for the palpable mass at the submandibular site.

A high‐grade epithelial carcinoma with solid architecture and some cystic areas was detected. Moreover, squamous cell differentiation and diffuse necrosis were described with isolated intracytoplasmic mucus‐containing elements.

On immunohistochemical analysis, tumor cells were positive for cytokeratins AE1/AE3 and P40, while negative for LCA, S‐100, CD‐117, GFAP, AML, chromogranin A, and synaptophysin. At the first step, high‐grade mucoepidermoid carcinoma and pleomorphic ex‐adenoma carcinoma were considered as differential diagnoses. Given the mucus‐containing elements, the former diagnosis was chosen as the final one. According to AJCC/UICC classification, tumor stage was pT2 pNx.

**FIGURE 2:**
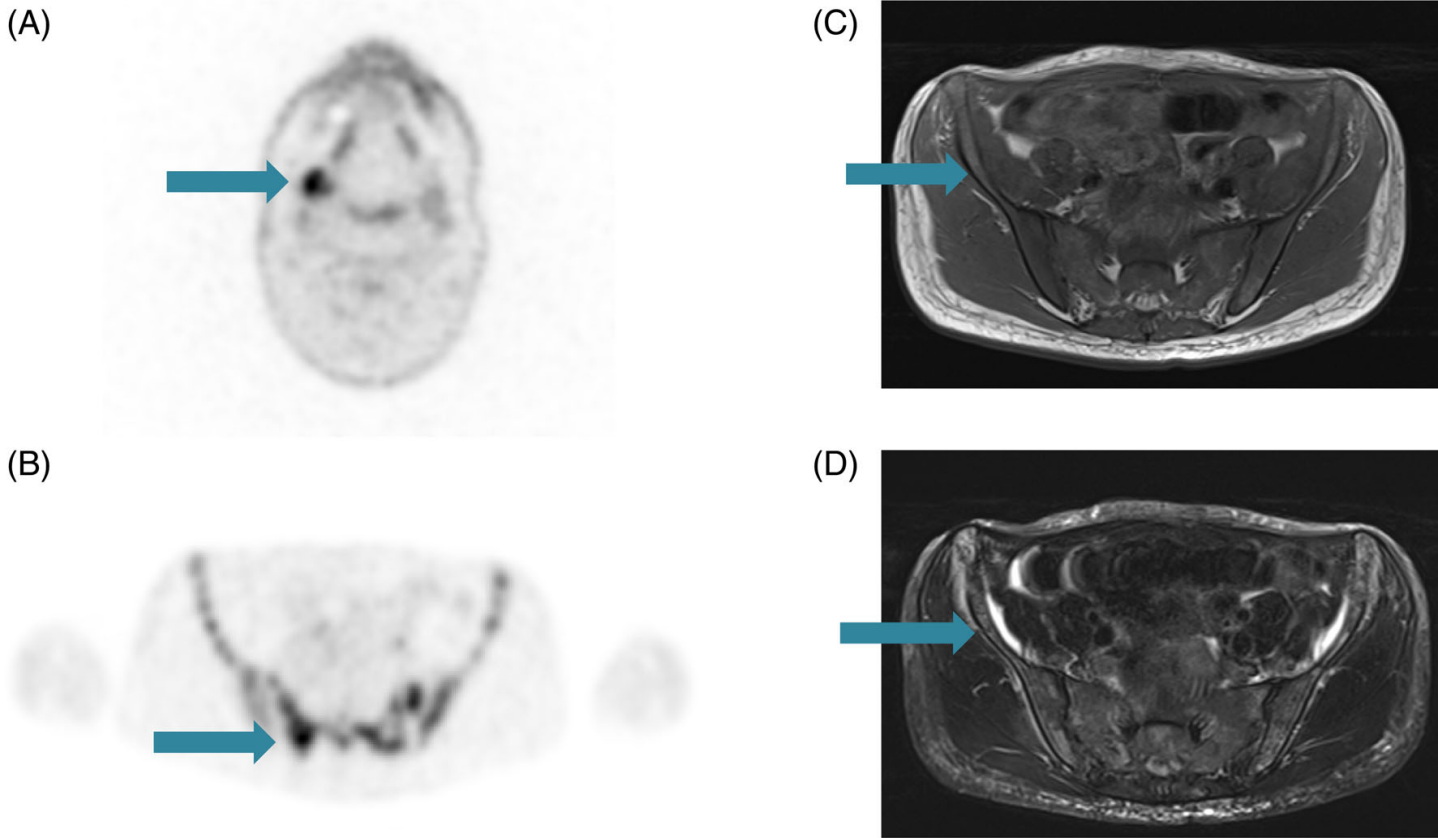
Fluorodeoxyglucose positron emission tomography (FDG PET) scan of primary tumor. Here it is possible to see the pathological hypercaptation at the right submandibular gland level (A). Bone lesions at metastatic diagnosis shown at FDG PET (B), and MRI scans in T1‐weighted (C) and T2‐weighted (D) sequences. The complete replacement of normal bone tissue is emphasized in these three images (B–D).

One month after surgery, restaging was performed with head and neck magnetic resonance imaging (MRI), and whole‐body computed tomography (CT), and fluorodeoxyglucose (FDG) positron emission tomography (PET) scans. No evidence of metastatic disease was detected at the CT scan, while the MRI showed a 22 cm solid alteration suspected of disease persistence in the right submandibular gland. A few subcentimeter neck lymph nodes were reported as well. FDG PET also indicated a pathological uptake at the same level (SUVm 14.2) (Figure 2A).

Then, a right radical neck dissection (levels I–V) was performed. Five regional lymph nodes were involved (3 massive and embolic lymph node metastases with extracapsular spread at level Ib, and 2 embolic and subcapsular regional metastases at level II).

No evidence of macroscopic disease persistence was found at the post‐operative head and neck MRI scan.

**FIGURE 3:**
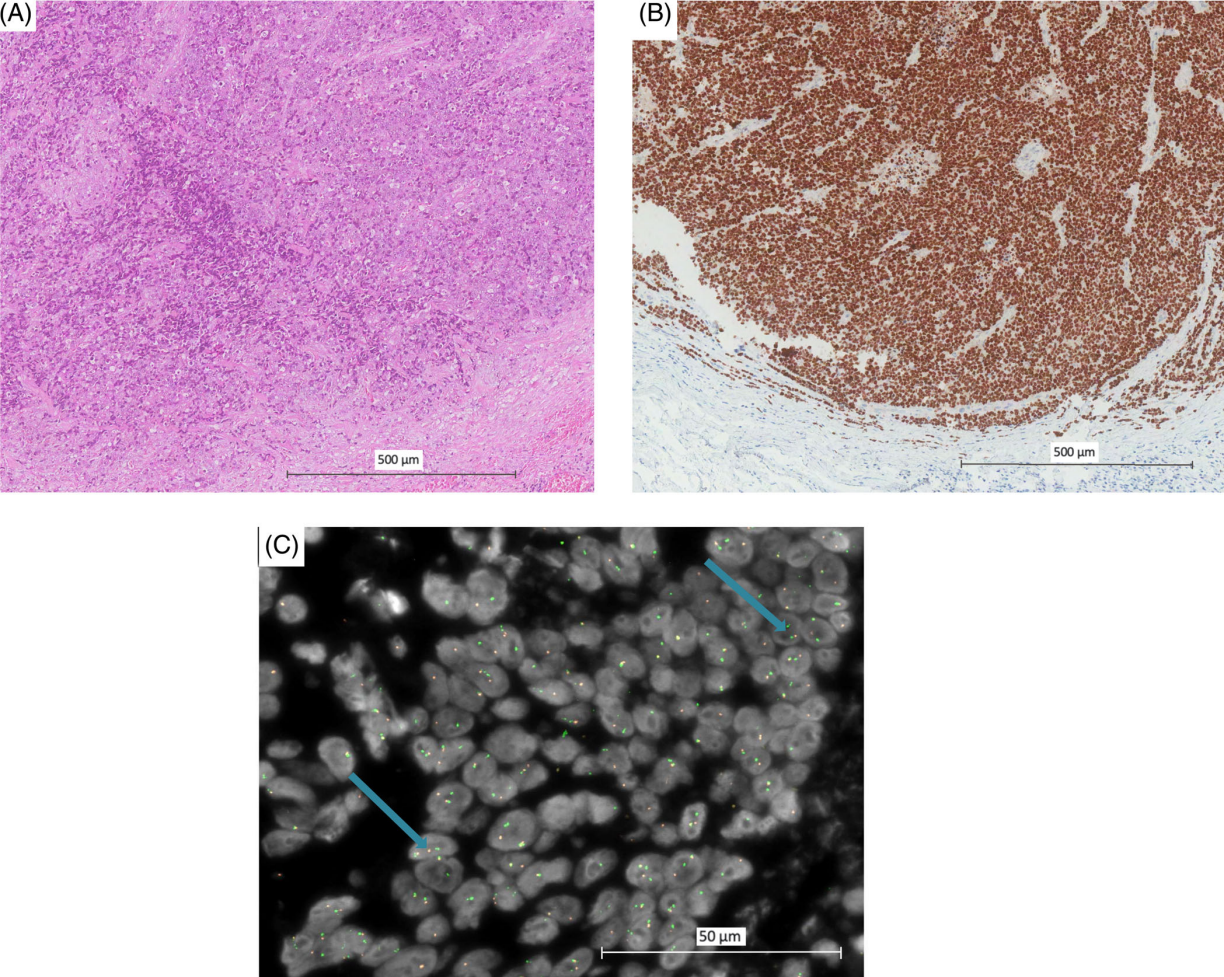
Morphological, immunohistochemical, and FISH NUTM1 features of the primary tumor. (A) Hematoxylin and eosin stain (100×); (B) NUT immunohistochemical expression (100×); (C) FISH NUTM1 break apart: separated green and orange signals indicate gene rearrangement (blue arrows). Counterstained with DAPI (1000×). In this figure, histological and immunohistochemical features of the neoplasm can be appreciated. In addition, in the third image, the genetic rearrangement of this malignancy is highlighted through the different colored signals.

In the same period, the patient sought a second opinion at our cancer center. Due to the rarity of salivary gland cancers and the uncertain diagnosis at the first pathologic report, a pathologic revision was performed. At the morphologic level, a poorly differentiated carcinoma with foci of abrupt keratinization was observed. At immunohistochemical analysis, tumor cells were positive for p63, p40, CK8/18, and negative for synaptophysin, actin 1A4, S100, CD99, NKX2.2, and androgen receptors. Given the aggressive clinical behavior, the poor differentiation of tumor cells, and the morphological features (notably the abrupt keratinization), immunohistochemistry for NUT was performed resulting in a strong and diffuse positivity. To confirm the diagnosis, a fluorescent in situ hybridization (FISH) of the NUTM1 gene was performed and identified an unbalanced translocation of the NUTM1 gene with loss of the 5′ derivative. NUT carcinoma was the final diagnosis (Figure 3).

Given the regional extension of the disease, the pathologic features (notably the massive and embolic node metastases with extracapsular spread), the well‐known aggressive behavior of NUTc, the young age of the patient, and the absence of comorbidities, the patient underwent adjuvant chemo‐radiation (intensity‐modulated radiotherapy for a total dose of 66 Gy in 33 fractions + concurrent chemotherapy with cisplatin, cumulative dose 200 mg/m^2^) with to increase her therapeutic chances. Indeed the approach to neoplasms of the head and neck involves intensifying post‐surgery treatments in high‐risk cases with adjuvant radiotherapy or a combined chemo‐radiation treatment.

Due to the high risk of disease recurrence, for the patient a strict follow‐up program was planned. Prior to the execution of the disease restaging with brain + head and neck MRI and with whole body FDG PET scans (planned 2 months after the conclusion of chemoradiation), there was an unexpected increase in fatigue, and the onset of widespread pain, more significant in the lumbosacral area. Although still able to be active for more than half the day, for the patient was difficult to lead a normal life.

No evidence of disease was found at loco‐regional level. However, a widespread metastatic disease was diagnosed: bone (entire spine, ribs bilaterally, homers, shoulder blades, clavicles, sternum, pelvis, and femurs), lung (bilaterally), extra‐regional lymph nodes (mediastinum), liver (two lesions) (Figure 2B–D).

Given the scarcity of effective treatments for metastatic NUTc, to evaluate a possible systemic treatment initiation with immunotherapy, assessment of TMB (0 mutations/MB), PDL1 (CPS = 10) and MSI (resulted stable – MSS), in addition to molecular assessment with NGS DNA were performed. No pathological mutations were detected at molecular analysis (Oncomine Comprehensive Assay Plus – analyzing more than 500 genes – technique was used).

Following a sudden decline in general condition (performance status—an international performance scale ranging from 0 to 5, where 0 stands for capable of normal activity and 5 for death—PS, ECOG 2), with uncontrolled pain, G2 fatigue, and G1 nausea, the patient was hospitalized.

A whole spine MRI was performed at the admission. Although spinal cord compression was excluded, a complete replacement of skeletal hematopoietic and adipose tissue was detected.

Palliative radiation with a single fraction (8 Gy) on L3–L5 was administered.

During the hospitalization, the performance status rapidly worsened (PS ECOG 3), and the lab tests showed a rapidly progressing hepatic failure (G3 alanine and aspartate aminotransferase, alkaline phosphatase, gamma‐glutamyltransferase increase), and pancytopenia (G3 anemia, G4 platelet count decreased; interpreted as myelophtisis). Patient was made aware of the poor prognosis and severity of the clinical conditions.

Given the further worsening of clinical conditions (PS ECOG 4) and lab tests, best supportive care was chosen as the only treatment that could be proposed to the patient.

The disease‐free interval between the end of chemoradiation and the onset of metastatic disease was 2.6 months.

The overall survival (OS) was 7.8 months from the diagnosis of primary disease, and 2.6 weeks from the first evidence of metastatic disease.

## 3 DISCUSSION

Based on the available evidence, only a minor proportion of NUTc are correctly diagnosed at disease presentation, while the majority still needs to be properly recognized.^3^ First of all, the difficult diagnosis is due to the NUTc rarity and its relatively recent definition. Indeed, the World Health Organization classification of head and neck tumors introduced this pathologic entity in its fourth edition in 2018.

The gold standard for diagnosis is the demonstration of NUT gene rearrangement by FISH or by polymerase chain reaction (PCR). Therefore, the difficulty of figuring out the indication to perform FISH or PCR may be an obstacle in the diagnostic path.^5^ Ideally, to avoid missing any diagnoses, they should be performed for any malignancy that is poorly differentiated, monomorphic, and does not stain for lineage‐specific markers.^5^

To further investigate the diagnostic challenge of NUTc and to compare the presented case with the current literature, in February 2023, we performed a literature review. We selected studies adopting the following selection criteria: (a) Case reports or case series articles; (b) patients affected by NUT carcinoma arising from major salivary glands; (c) no time filters were applied.

All articles were searched using the combination of the keywords “NUT carcinoma” and “salivary gland” or “submandibular” or “parotid” or “sublingual” in the titles/abstract of publications using the advanced research mode of PubMed.

We found 19 cases of NUTc arising from major salivary glands reported in the literature, with a timeframe ranging from 2009 to 2023.^12^, ^13^, ^14^, ^15^, ^16^, ^17^, ^18^, ^19^, ^20^, ^21^, ^22^, ^23^, ^24^, ^25^, ^26^, ^27^ (Tables 1 and 2, Supplementary Table S1).

**TABLE 1:** Clinical features of the case reports published in the literature.

Article	Year	Age	Gender	Site	Early symptoms
Current case	2023	39	F	Submandibular gland	Mass
Ziai et al.	2010	15	M	Submandibular gland	Mass and pain
Moreno et al.	2022	49	M	Submandibular gland	Mass and pain
Cho et al.	2017	29	F	Submandibular gland	Mass and pain
Wang et al.	2021	9	M	Submandibular gland	Mass and pain
Andreasen et al.	2015	40	F	Sublingual gland	Swelling and meal related pain
Storck et al.	2017	9	M	Sublingual gland	Mass, fever, vomiting, indigestion
Seim et al.	2017	26	M	Sublingual gland	Neck pain, swelling, otalgia
Den Bakker et al.	2009	15	M	Parotid	Mass
Park et al.	2014	12	M	Parotid	Mass
Vulsteke et al.	2016	32	M	Parotid	Mass and swelling
Klijanienko et al.	2016	21	F	Parotid	Mass
Agaimy et al.	2010	39	F	Parotid	Mass
Agaimy et al.	2015	35	M	Parotid	Mass
Agaimy et al.	2017	55	F	Parotid	Mass
Saik et al.	2020	34	F	Parotid	Mass
Esteves et al.	2020	34	M	Parotid	Mass
Lemelle et al.	2017	21	F	Parotid	NA
Shujuan Fu et al.	2023	36	F	Parotid	Mass

**TABLE 2:** Previous misdiagnoses and patient outcomes of the case reports published in the literature.

Article	Misdiagnosis	Original diagnosis	Death	Disease free interval (DFI) (months)	Overall survival (OS) (months)
Current case	Yes	High‐grade mucoepidermoid carcinoma	Yes	2.6	7.8
Ziai et al.	No		No	14.0^a^	
Moreno et al.	No		No	4.0^a^	
Cho et al.	Yes	Metastatic carcinoma with sarcomatoid features and myxoid matrix formation	No	20.0^a^	
Wang et al.	No		Yes		8.0
Andreasen et al.	Yes	Poorly differentiated squamous cell carcinoma (SCC) with basaloid features	Yes	1.0	5.5
Storck et al.	No		No	72.0^a^	
Seim et al.	Yes	Undifferentiated carcinoma^c^	Yes		4.0
Den Bakker et al.	Yes	Poorly differentiated carcinoma^c^	No	7.0^a^	
Park et al.	Yes	Poorly differentiated squamous carcinoma	Yes		24.0
Vulsteke et al.	Yes	Myoepithelial carcinoma with neuro‐endocrine features	Yes		NA
Klijanienko et al.	Yes	Poorly differentiated adenoid cystic carcinoma, then high grade lymphoma	Yes	3.0	5.0
Agaimy et al.	Yes	High‐grade mucoepidermoid carcinoma	Yes	3.0	7.0
Agaimy et al.	Yes	Undifferentiated large cell carcinoma	No	3.0^b^	
Agaimy et al.	Yes	Undifferentiated tumor^c^	Yes	3.0	7.0
Saik et al.	Yes	High‐grade neuroendocrine carcinoma	Yes	1.0	6.0
Esteves et al.	No		No	4.0^a^	
Lemelle et al.	NA		Yes		3.0
Shujuan Fu et al.	No		Yes	6.0	24.0

^a^ Not evidence of disease (NED) at the time of article publishing.

^b^ Palliative therapy at the time of article publishing.

^c^ No more information about first wrong diagnosis could be detected from the case report.

Median age was 30.5 years (range 9–55), and most patients (10) were men. The most frequent primary tumor site was the parotid (10), and the least the sublingual glands (3). All patients at the onset of the disease described “mass sensation” of the tumor‐affected area as their first symptom; six complained of concomitant pain, and one also reported vomiting and indigestion.

Most cases (13) had regional lymph node involvement at diagnosis, and distant metastases were observed upfront in two patients. In cases developing distant metastases, the most frequent distant sites were bone (10), followed by liver (6), and lung (4). Meningeal (2) and brain (1) involvement were also observed. Nearly all patients underwent surgery as upfront treatment, and nine received adjuvant concomitant chemoradiation. Unfortunately, the majority rapidly developed a disseminated disease requiring palliative systemic treatments. In this setting, platinum salts were the most frequently used agents, while immune checkpoint inhibitors were employed in two cases.

At least in 12 of the identified cases, a misdiagnosis was made at the beginning of the therapeutic pathway, leading to a delay in initiating treatment. The initial diagnosis was undifferentiated/squamous cell carcinoma in 7 cases, high‐grade mucoepidermoid carcinoma in 2, carcinoma with endocrine differentiation in 2. Seven patients were alive at the time of article publishing, in one case without evidence of disease at 72 months. Among patients with a post‐treatment follow‐up, median OS was 7.0 months, consistent with data from the literature.^3^

With the exception of the current case, NGS molecular analysis was assessed in two cases only, with no relevant results that resulted in targeted therapy. Microsatellite status, CPS, or TMB were assessed in only one of the cited case reports.

The WHO classification of head and neck tumors defines NUTc as a “poorly differentiated carcinoma, often with evidence of squamous differentiation”, indeed the most common misdiagnoses are poorly differentiated carcinoma and poorly differentiated squamous carcinoma, as underlined in Table 2.^5^, ^12^, ^13^, ^14^, ^15^, ^16^, ^17^, ^18^, ^19^, ^20^, ^21^, ^22^, ^23^, ^24^, ^25^, ^26^, ^27^

Finally, an important role is played by the pathology reference centers. As demonstrated by the current case and those reported in the literature, a misdiagnosis in a peripheral center is quite common. At the same time, the correct diagnosis is often reached only after a second opinion in a specialized high‐volume clinical institution.^12^, ^13^, ^14^, ^15^, ^16^, ^17^, ^18^, ^19^, ^20^, ^21^, ^22^, ^23^, ^24^, ^25^, ^26^, ^27^, ^28^

In addition to a delay in initial diagnosis, another problem in rare cancers is the risk of receiving inadequate curative treatment. Moreover, as a consequence of the lack of both academic and industrial sponsors and of the low prevalence of the malignancy, few clinical trials are available for rare cancer patients, although being a heterogeneous population to be cautiously analyzed as a single group.^28^ Hence, so a reduced therapeutic choice is available for these subjects.^29^

To increase access to accurate pathological and molecular diagnosis of rare cancers and to offer optimal treatments, virtual international networks connecting patients and health care providers have been established.^30^ In Europe, the OS rate for rare cancers is still lower than for more common types of cancer. From this perspective, the European reference network for rare cancers aims to increase coordination between centers in different countries, implement guidelines, and facilitate patient access in referral centers.^30^

A high level of expertise is the only way to improve the timing of diagnosis, treatment, and prognosis of NUTc and rare cancers.

Due to the rarity of the malignancy and its high aggressiveness, there is still no standard treatment for NUTc. A study conducted in NUTc arising of the head and neck district stated that the only patients experiencing a prolonged survival were those who achieved complete remission after surgery.^3^ Moreover, the role of postoperative therapy and its possible positive effect on progression‐free (PFS) and OS is still unclear, even if better results in highly aggressive head and neck malignancies are achieved with multimodality treatments.^3^

Therefore, considering adjuvant chemo‐radiotherapy treatment in operable NUTc may be a reasonable choice.

As underlined by our review, in the field of medical systemic treatments, multiple chemotherapeutics have been tried with poor results, including doxorubicin, carboplatin, cisplatin, cyclophosphamide, vincristine, etoposide, actinomycin D, vinorelbine, vinblastine, paclitaxel, docetaxel, 5‐fluorouracil, bleomycin, ifosfamide, gemcitabine, and BET inhibitors.^31^ In the metastatic setting, palliative chemotherapy remains the only possible choice.

Molecular profiling using NGS and a PDL‐1 status assessment should be considered to find potential therapies.

in clinical practice and increase the knowledge about the biology of NUTc. Indeed, there are scant data in the literature regarding molecular profiling of NUTc and none in those of the head and neck. Xie et al. analyzed with NGS 10 patients affected by NUTc arising from the lung to explore its genomic landscape. The most common alterations identified were P53, PIK3CA, AUTS2, ITIH2, and CDKL5.^32^

In the field of immune‐oncology, there is currently no evidence of the possible use of immunotherapy in clinical practice, and we lack literature data to support its activity. In this scenario, the activity and safety of immune checkpoint inhibitors are under study in lung NUTc (e.g., atezolizumab in pretreated patients with advanced non‐small cell lung cancer with rare histologic subtypes, including NUTc).^33^

Finally, as reported in a recent case report of a sinonasal NUTc, proton therapy could be a valid approach, particularly for elderly and frail patients.^34^

Describing this case, in addition to the relevance of its rarity, allowed us to analyze the existing literature, primarily emphasizing the fundamental relevance of referral centers to have correct diagnoses and reflecting on how to improve the diagnostic and treatment pathway of these patients. Collecting cases in common databases and networking between cancer centers are fundamental. In this setting, a specific registry is open in the United States,^35^ and more recently, the observational clinical registry of the European Reference Network on Rare Adult Solid Cancers has been delivered.^36^

## 4 CONCLUSIONS

We presented the case of a patient with a NUTc arising in the submandibular gland aiming to increase knowledge of this rare cancer. We also reviewed the cases of major salivary gland NUTc published so far.

Data from our review are consistent with the literature, stating a median OS of 7.0 months, underlying the high aggressiveness of NUTc and the lack of a standard therapy pathway.

The only patients with a prolonged survival are those who achieved complete remission after surgery.

Based on the described case, the data collected and the rarity of the disease:

1. The access to referral centers is crucial to guarantee a prompt and correct diagnosis. Virtual international cancer networks connecting patients and health care providers have been established, also aiming to strengthen roadmaps to high expertise centers;
2. At least in the advanced phase of the disease, molecular profiling and assessment of immune biomarkers (e.g., PD‐L1, TMB) may be suggested to provide NUTc patients with personalized therapeutic options, and to increase the clinical and biological knowledge of this rare and aggressive cancer;
3. At the time we are writing, there is no evidence of activity of immunotherapy in NUTc, although one clinical study is still ongoing.^32^

## AUTHOR CONTRIBUTIONS

**Simone Rota:** Writing – original draft (lead); writing – review and editing (lead). **Pasquale Quattrone:** Conceptualization (equal); data curation (equal); visualization (equal). **Giovanni Centonze:** Visualization (equal). **Gianpaolo Dagrada:** Data curation (equal). **Arianna Ottini:** Funding acquisition (equal); investigation (equal); resources (equal). **Elena Colombo:** Funding acquisition (equal); resources (equal). **Imperia Nuzzolese:** Formal analysis (equal); resources (equal). **Giuseppina Calareso:** Visualization (equal). **Marzia Franceschini:** Data curation (equal). **Nicola Alessandro Iacovelli:** Resources (equal). **Federica Perrone:** Data curation (equal). **Elena Tamborini:** Resources (equal). **Stefano Cavalieri:** Methodology (lead); project administration (lead); supervision (lead); writing – original draft (equal); writing – review and editing (equal).

## CONFLICT OF INTEREST STATEMENT

The authors have stated explicitly that there are no conflicts of interest in connection with this article.

## ETHICS STATEMENT

Hereby, I, Simone Rota, consciously assure that for the manuscript—NUT carcinoma of the submandibular gland: A case report and literature review—the following is fulfilled: This material is the authors' own original work, which has not been previously published elsewhere. The paper is not currently being considered for publication elsewhere. The paper reflects the authors' own research and analysis in a truthful and complete manner. The paper properly credits the meaningful contributions of co‐authors and co‐researchers. The results are appropriately placed in the context of prior and existing research. All sources used are properly disclosed. All authors have been personally and actively involved in substantial work leading to the paper, and will take public responsibility for its content.

## CONSENT STATEMENT

Since no identifiable information is presented in the article and the patient deceased in 2022, informed consent could not be obtained. However, given that the patient accessed to a tertiary cancer center which is dedicated to the clinical management and the research on cancer patients, in agreement with the Italian laws, we deemed it implicit that the patient herself agreed with the use of her unidentifiable data for research purposes.

## Supporting information


**Supplementary Table 1.** Diagnosis and treatments of the case reports published in the literature.

## Data Availability

Authors confirm that data supporting this case report and the associated literature review are available within the article and its supplementary material.
