# Supplementary material for: NUT carcinoma of the submandibular gland: A case report
Source: Cancer Rep (Hoboken). 2023 Sep 8;6(12):e1900. doi: 10.1002/cnr2.1900 (PMC10728538; doi:10.1002/cnr2.1900)
Supplement: Supplementary file 1 — Supplementary Table 1. Diagnosis and treatments of the case reports published in the literature. [file CNR2-6-e1900-s001.docx]

| **Article** | **Lymph node involvement**  **at diagnosis** | **Metastasis at diagnosis** | **Metastasis sites at diagnosis** | **Sites of subsequent metastasis** | **Type of treatment** | **Drug used** |
| --- | --- | --- | --- | --- | --- | --- |
| Current case | No | No |  | bone, lung, liver, extraregional lymph nodes | surgery + concomitant chemo-radiation (CTRT) | Cisplatin (CDDP) |
| Ziai et al. | Yes | No |  |  | surgery + RT |  |
| Moreno et al. | Yes | No |  |  | surgery + CTRT + palliative ct | Carboplatin (CBCDA) + paclitaxel |
| Cho et al. | Yes | Yes | liver |  | surgery + CTRT + palliative CT | Docetaxel, CDDP |
| Wang et al. | Yes | Yes | liver, brain, bone. | leptomeninges | Chemotherapy (CT) with partial response (PR), then surgery and again palliative CT | Vorinostat + paclitaxel + CDDP,  ifosfamide + vorinostat,  vorinostat + cyclophosphamide + topotecan |
| Andreasen et al. | No | No |  | bone | surgery + CT | paclitaxel + capecitabine |
| Storck et al. | Yes | No |  |  | CTRT | vincristine + adriamicina + ifosfamide (VAI) cisplatino + adriamicina + ifosfamide (PAI) |
| Seim et al. | No | No |  | bone, lung, meninges | CTRT | docetaxel + CDDP |
| Den Bakker et al. | Yes | No |  |  | surgery + CTRT | CDDP + ifosfamide + doxorubicine + etoposide + vincristine |
| Park et al. | Yes | No |  |  | surgery + CTRT | NA |
| Vulsteke et al. | No | No |  | bone, soft tissue | surgery + RT, then palliative CT | CCDP + doxorubicine + ifosfamide for two cycles, then CDDP + cyclophosphamide + doxorubicine |
| Klijanienko et al. | Yes | No |  | liver, soft tissue | surgery + RT |  |
| Agaimy et al. | Yes | No |  | bone, lung, extraregional lymph nodes | surgery + CTRT, then palliative RT and a BET inibitor | BET inhibitor |
| Agaimy et al. | Yes | No |  | bone | surgery + palliative CT | NA |
| Agaimy et al. | Yes | No |  | bone, liver, lung | surgery + CTRT |  |
| Saik et al. | Yes | No |  | bone | surgery + RT, then palliative immunotherapy (IT) | Pembrolizumab |
| Esteves et al. | No | No |  |  | surgery + CTRT |  |
| Lemelle et al. | Yes | No |  |  | surgery + RT, then palliative CT | Platinum compounds |
| Shujuan Fu et al. | No | No |  | Liver,lymph nodes | surgery + CTRT, then palliative CT and TKI inhibitors | Vincristine + epirubicin +cyclophosphamide (VAC)  ifosfamide + etoposide  BET inhibitor + lenvatinib + sintilimab |

Supplementary Table. Diagnosis and treatments of the case reports published in the literature
